# *POMFinder*: identifying polyoxometallate cluster structures from pair distribution function data using explainable machine learning

**DOI:** 10.1107/S1600576723010014

**Published:** 2024-02-01

**Authors:** Andy S. Anker, Emil T. S. Kjær, Mikkel Juelsholt, Kirsten M. Ø. Jensen

**Affiliations:** a Department of Chemistry and Nano-Science Center, University of Copenhagen, 2100 Copenhagen Ø, Denmark; b Department of Materials, University of Oxford, Parks Road, Oxford, Oxfordshire OX1 3PH, United Kingdom; Australian Nuclear Science and Technology Organisation, Lucas Heights, Australia

**Keywords:** computational modelling, machine learning, polyoxometallate clusters, *POMFinder*

## Abstract

Characterization of a material structure with pair distribution function (PDF) analysis typically involves refining a structure model against an experimental data set, but finding or constructing a suitable atomic model for PDF modelling can be an extremely labour-intensive task, requiring carefully browsing through large numbers of possible models. Presented here is *POMFinder*, a machine learning (ML) classifier that rapidly screens a database of structures, here polyoxometallate (POM) clusters, to identify candidate structures for PDF data modelling. The approach is shown to identify suitable POMs from experimental data, including *in situ* data collected with fast acquisition times. This automated approach has significant potential for identifying suitable models for structure refinement to extract quantitative structural parameters in materials chemistry research. *POMFinder* is open source and user friendly, making it accessible to those without prior ML knowledge. It is also demonstrated that *POMFinder* offers a promising modelling framework for combined modelling of multiple scattering techniques.

## Abstract 2

Machine learning is used to analyse pair distribution functions obtained from polyoxometallate clusters. The new classifier *POMFinder* can rapidly screen a database of structures and identify which model is most suitable for describing the experimental data.

## 1. Introduction

The continued development of increasingly bright synchrotron and neutron facilities means that scattering and spectroscopy data can now be measured at impressive speeds (Wang *et al.*, 2018 ▸; Dong *et al.*, 2021 ▸; Pacchioni, 2019 ▸). Hundreds of gigabytes or even terabytes of data are now commonly collected in each experiment, each data set containing thousands or millions of individual measurements. With this amount of data, it is an enormous challenge to work through each data set manually, and the development of automated methods for data analysis is thus becoming more and more necessary (Dong *et al.*, 2021 ▸; Chen *et al.*, 2021 ▸; Choudhary *et al.*, 2022 ▸).

For many X-ray- and neutron-based scattering techniques such as small-angle scattering, powder diffraction and total scattering with pair distribution function (PDF) analysis, data analysis is often done through least-squares optimization (Pedersen, 1997 ▸; Rietveld, 1969 ▸; Chepkemboi *et al.*, 2022 ▸). Here, structure models found in *e.g.* structure databases are used to simulate data, which are then refined against experimental data. This allows the extraction of quantitative structural parameters. This approach can, in principle, be automated by *e.g.* testing entire databases of structures against experimental data sets (Banerjee *et al.*, 2020 ▸; Yang *et al.*, 2020 ▸; Aimi & Fujimoto, 2020 ▸; Christiansen *et al.*, 2020*b* ▸). However, least-squares fitting algorithms are computationally expensive, which makes them unsuited for automatically identifying and refining structures from experiments with many data sets (Wang *et al.*, 2018 ▸). Consequently, identifying structural models is currently a bottleneck for modelling large quantities of scattering data.

In this study, we present a tree-based machine learning (ML) classifier that identifies a chemical structure from a PDF in less than one second, enabling high-throughput database screening. The PDF here refers to the reduced pair distribution function *G*(*r*), which represents a histogram of real-space interatomic distances and can be used to identify atomic arrangements in materials. *G*(*r*) is obtained by Fourier transforming the total scattering structure function *S*(*Q*), which is the set of corrected and normalized total scattering data (Egami & Billinge, 2012 ▸),

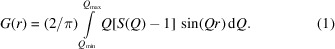

Here, *Q* is the magnitude of the scattering vector [*Q* = (4π/λ)× sin(θ/2), where θ is the scattering angle and λ is the wavelength of the incident radiation], while *r* is the interatomic distance. The *Q* range used for modern total scattering experiments ranges from *Q*_min_ = 0.1–1 Å^−1^ to *Q*_max_ = 15–30 Å^−1^.

In recent years PDF analysis has been shown to be a powerful technique for characterization of disordered materials (Christiansen *et al.*, 2020*b* ▸; Yang *et al.*, 2013 ▸; Billinge & Kanatzidis, 2004 ▸; Keen & Goodwin, 2015 ▸), amorphous materials (Christiansen *et al.*, 2020*a* ▸; Juelsholt *et al.*, 2021 ▸; Bennett & Cheetham, 2014 ▸), clusters in solution (Anker *et al.*, 2021 ▸; Jensen *et al.*, 2016 ▸; Szczerba *et al.*, 2021 ▸) and nanomaterials (Billinge & levin, 2007 ▸; Cooper *et al.*, 2020 ▸) where conventional crystallographic approaches are challenged (Billinge & Kanatzidis, 2004 ▸; Keen & Goodwin, 2015 ▸).

PDFs are usually analysed by fitting a reasonable starting model to the experimental PDF using dedicated software such as *PDFgui* (Farrow *et al.*, 2007 ▸), *DiffPy-CMI* (Juhás *et al.*, 2015 ▸), *DISCUS* (Proffen & Neder, 1997 ▸, 1999 ▸) or *TOPAS* (Coelho, 2018 ▸). In some cases, for example for well characterized crystalline materials, identifying a starting model for structural refinements is easily done. In other cases, finding or constructing a good initial atomic model for modelling the PDF can be an extremely labour-intensive task, requiring carefully browsing through large numbers of possible starting models. However, we and others have shown that ML methods such as neural networks and tree-based ML have much potential to improve the speed of PDF analysis (Anker *et al.*, 2020 ▸, 2022 ▸, 2023 ▸; Liu *et al.*, 2019 ▸; Kjær *et al.*, 2023 ▸; Kløve *et al.*, 2023 ▸; Skjaervø *et al.*, 2023 ▸; Magnard *et al.*, 2022 ▸). ML has, for example, been used to identify crystallographic space groups from PDFs (Liu *et al.*, 2019 ▸), to extract structural motifs (Anker *et al.*, 2022 ▸; Skjaervø *et al.*, 2023 ▸; Magnard *et al.*, 2022 ▸) and to determine the structure of small metallic nanoparticles (Anker *et al.*, 2020 ▸; Kjær *et al.*, 2023 ▸).

We here use a tree-based ML classifier to identify the structure of polyoxometallate (POM) clusters in solution on the basis of a PDF. POM clusters are a family of large polyanion clusters mostly constructed of [*M*O_6_] octahedra, where *M* is often Mo, W, V or Nb (Gumerova & Rompel, 2018 ▸, 2020 ▸; Miras *et al.*, 2012 ▸; Long *et al.*, 2010 ▸). POMs have been extensively studied due to both their rich chemistry and their many applications, *e.g.* in molecular magnets, as catalysts for water splitting, as conductors or in medicine (Gumerova & Rompel, 2018 ▸, 2020 ▸; Miras *et al.*, 2012 ▸; Long *et al.*, 2010 ▸). Furthermore, it has been shown that the formation of metal oxide crystals can be dependent on the structure of the POM cluster, which has a huge impact on the formation mechanism (Christiansen *et al.*, 2020*b* ▸; Juelsholt *et al.*, 2019 ▸). While POMs have so far been mainly studied in the crystalline form, PDF analysis allows POM structure studies in solution, which paves the way for a new understanding of their chemistry.

The ML model, which we refer to as *POMFinder*, has been trained on simulated PDFs from 443 POM clusters, cut out of crystal structures containing POMs obtained from the Crystallography Open Database (COD; Gražulis *et al.*, 2018 ▸) and the Inorganic Crystal Structure Database (ICSD; Allen *et al.*, 1987 ▸). *POMFinder* allows identification of POM structures from PDFs and has an accuracy of 94.0% on simulated data within the first prediction. It also shows good performance on experimental PDF data. We use SHapley Additive exPlanations (SHAP; Lundberg & Lee, 2017 ▸; Lundberg *et al.*, 2020 ▸) analysis to understand the predictions of *POMFinder*. With SHAP analysis, we can calculate the contribution of each input feature in the ML model to its predictions. Using SHAP analysis on simulated PDFs from X-rays (xPDF), neutrons (nPDF) and electrons (ePDF), we show that *POMFinder* learns trends corresponding to the scattering power of the different elements in the POMs and uses this information in its predictions. Finally, we show that the method can be extended to use data jointly from multiple scattering techniques instead of analysing the data separately, comparable to the ‘complex modelling’ approach (Billinge & Levin, 2007 ▸). We use simulated xPDF, small-angle X-ray scattering (SAXS) data, nPDF and ePDF, as well as combinations of the above data sets. A common problem of complex modelling is to weight the data sets (Anker *et al.*, 2021 ▸; Juhás *et al.*, 2015 ▸; Krayzman *et al.*, 2008 ▸), but this is not necessary when using ML to identify the structural model.

## 2. Construction of the POM database and training of the *POMFinder* model

We aim to create an ML model that can quickly and efficiently match an atomic POM structure to experimental data. We have chosen to focus on X-ray PDF data as the structural characterization technique and on POMs as the structures of interest. In principle, however, the data set can be any information that can be modelled using an atomistic model and represented in a tabular data format. The goal is *not* to have an algorithm that can output the perfect model to all experimental data every time with no user input. Instead, the successful ML model can filter out all bad models and give the user a handful of models which can be used for further analysis.

**Figure 1:**
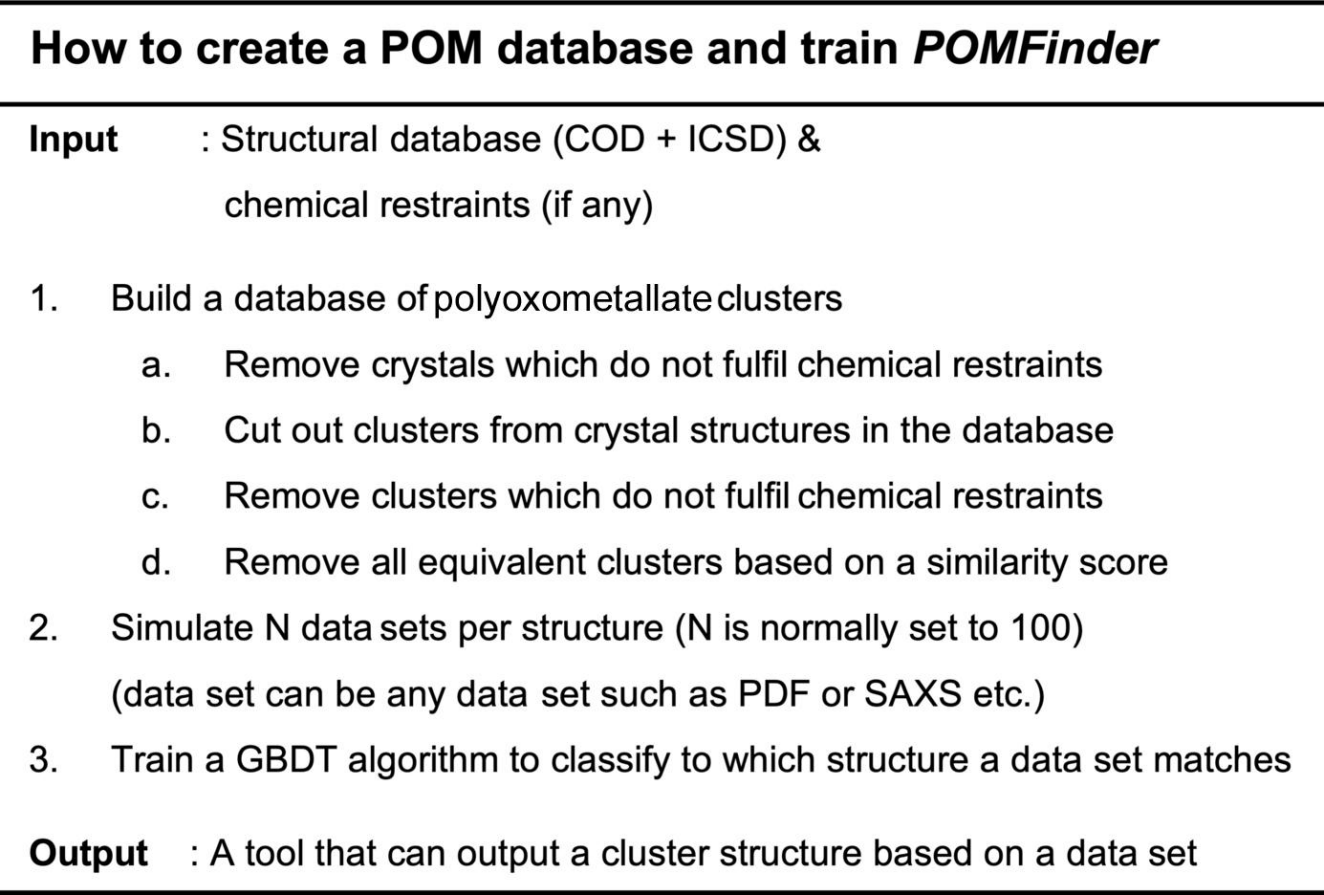
Pseudo-code describing how to create POM clusters from a CIF database and how to train *POMFinder*. A POM database is built from the ICSD and COD by cutting out clusters from all crystal structures with chemical compositions similar to POM clusters in solution (step 1) (Gumerova & Rompel, 2020 ▸). A number of PDFs are simulated for each POM cluster using various parameters (step 2). These PDFs are then used to train a GBDT model for classifying the corresponding structure from a PDF (step 3).

A pseudo-code of how to create the POM database and train *POMFinder* can be seen in Fig. 1 ▸. The structural database of POM clusters is built from crystallographic information files (CIFs) obtained from both the COD and ICSD using chemical restraints appropriate for POM clusters, as discussed below. Afterwards, a number (*N*) of PDFs are simulated for each POM structure with varying simulation parameters (*Q*_min_, *Q*_max_, an instrumental damping parameter *Q*_damp_ and an isotropic atomic displacement parameter ADP). The *Q* range used in the PDF [equation (1)] affects the *r* resolution of the PDF, and the limited *Q* range (*Q*_min_–*Q*_max_) creates termination ripples in the PDF (Egami & Billinge, 2012 ▸). Therefore, automated PDF data analysis must be applicable across various *Q* ranges. The parameters are varied using Latin hypercube sampling (Bouhlel *et al.*, 2019 ▸), and a gradient-boosting decision tree (GBDT) model (Chen & Guestrin, 2016 ▸; https://xgboost.readthedocs.io/en/stable/index.html#) is trained to classify which POM structure best matches the input data. In the following sections we will elaborate on this process.

### 2.1. Building a database of polyoxometallate clusters

**Figure 2:**
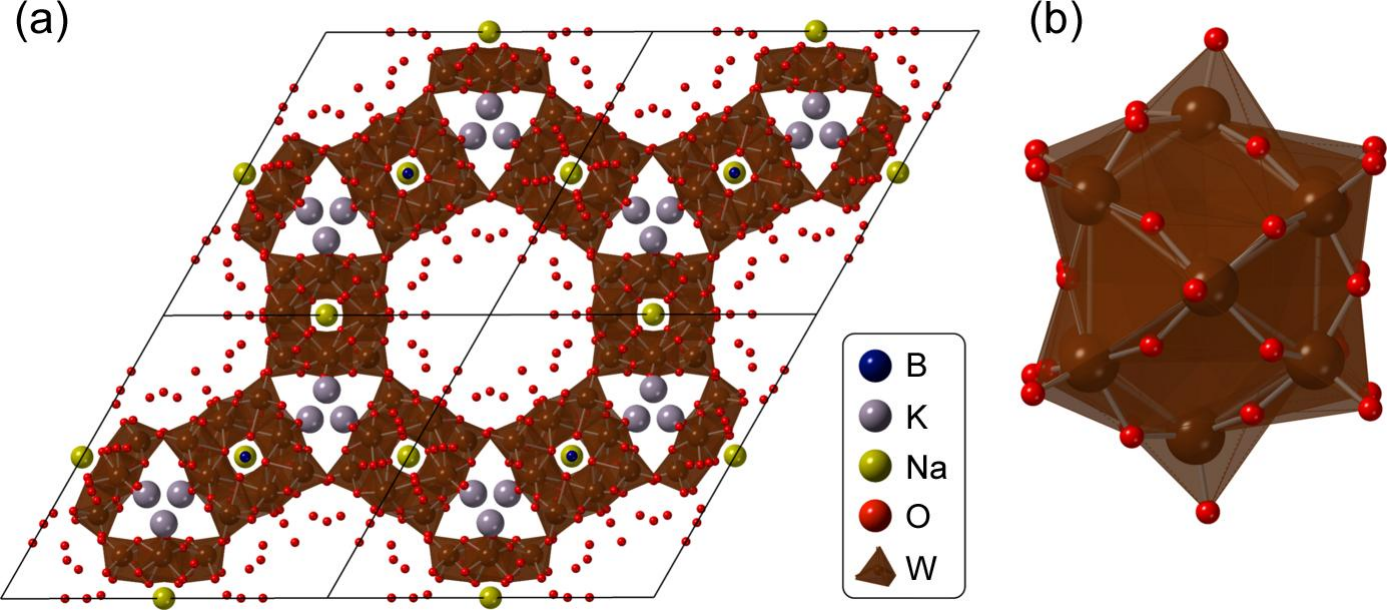
A POM cluster cut out from a crystal structure. (*a*) The crystal structure of K_2_NaH_2_[BW_12_O_40_]·12H_2_O (Han *et al.*, 2012 ▸) and (*b*) the corresponding POM cluster. W is shown in brown, O in red, Na in yellow, B in blue and K in grey. H is omitted for clarity.

The COD and ICSD databases contain hundreds of thousands of CIFs. When building our database, we first screened for CIFs with the same metal–oxygen ratios as described in a comprehensive review of POM clusters in solution by Gumerova & Rompel (2020 ▸). This restrained the database to 56 different metal–oxygen ratios, yielding 1281 CIFs. Clusters were then cut out of the CIFs by creating a 2 × 2 × 2 unit cell of the crystal and extracting all clusters of atoms not bonded to other atoms in the structure. Some clusters span more than a single unit cell, so to capture the complete POM cluster, a 2 × 2 × 2 unit cell was needed. Next, all isolated clusters that did not fulfil the chemical restraints (the 56 different metal–oxygen ratios) were removed. Fig. 2 ▸ illustrates an example of a cluster that was cut out of a crystal built from Keggin polyoxoanions, K_2_NaH_2_[BW_12_O_40_]·12H_2_O (Han *et al.*, 2012 ▸).

This procedure yielded 969 potential polyoxometallate clusters. The simulated PDFs’ Pearson correlation coefficients (PCCs) were used to remove similar structures from the database (Kjær *et al.*, 2022 ▸). The PDFs were compared iteratively by simulating a PDF of the first and second clusters with the parameters given in Section A in the supporting information and comparing their absolute PCCs. The PCC is a measure from −1 to 1 of how linearly correlated two continuous data sets are, where −1 represents inverse data sets and 1 represents identical data sets. If the absolute PCC was higher than 0.99, the second cluster was not included in the database. The third cluster was then compared with the first and second clusters by the same procedure and so on. The value of 0.99 was defined by manually inspecting the structures, their corresponding PDFs and the PCCs. Examples of three structures, their corresponding simulated PDFs and the PCCs can be seen in Section A in the supporting information. This process was performed with all 969 structures, yielding 443 unique structures. We note here that it is not guaranteed that the clusters are perfectly cut out of the crystal structure, which makes it important for the user to inspect the results of *POMFinder* and establish whether they make chemical sense.

### 2.2. Simulation of PDFs from the POM structures and training process of *POMFinder*

For each structure, a number (*N*) of PDFs were simulated with a broad range of instrumental parameters sampled using Latin hypercube sampling (Bouhlel *et al.*, 2019 ▸.) The simulations were done using *DiffPy-CMI* (Juhás *et al.*, 2015 ▸). The parameters are *Q*_min_, *Q*_max_, *Q*_damp_ and the ADPs. Section B in the supporting information gives the range of simulation parameters for the PDF data. The PDFs are normalized to have *G*(*r*)_max_ = 1, and all intensities up to *r* = 1 Å are set to 0 since the POM clusters are unlikely to have atomic distances that contribute to the signal in this range of the PDF. While this normalization is vital for aligning the training set with experimental PDFs, we use the PDFs in their unmodified form for fitting procedures and for all visual representations within this paper.

An example of an experimental PDF before and after normalization is shown in Section B in the supporting information.

The simulated data sets and their corresponding instrumental parameters (*Q*_min_, *Q*_max_, *Q*_damp_ and ADPs) are input in a GBDT model. The GBDT algorithm used is *XGBoost* with default parameters, except for the learning rate, which was set to 0.3, and the early stop criterion of five rounds without improvement (Chen & Guestrin, 2016 ▸; https://xgboost.readthedocs.io/en/stable/index.html#). The problem is a 443 class classification problem with an input of 443 × *N* simulated PDFs. For each structure, two of the 100 simulated PDFs are randomly chosen and set aside during the training of the model and later used as validation and test sets. The validation set is used to validate when the GBDT model has converged.

The loss curve (multiclass log loss; https://scikit-learn.org/stable/modules/generated/sklearn.metrics.log_loss.html) is plotted in Section C in the supporting information, which shows that the model can predict the training data with 100% accuracy, while the validation data are predicted with a small loss. The concluding accuracy of the model can be determined on the test set, which are data on which the model has not been trained or validated, *i.e.* comparable to how *POMFinder* can be used for experimental data. When *POMFinder* is trained on 100 PDFs for each structure, the accuracy on the test set is 94.0% according to test set predictions.

## 3. Use of *POMFinder*

**Figure 3:**
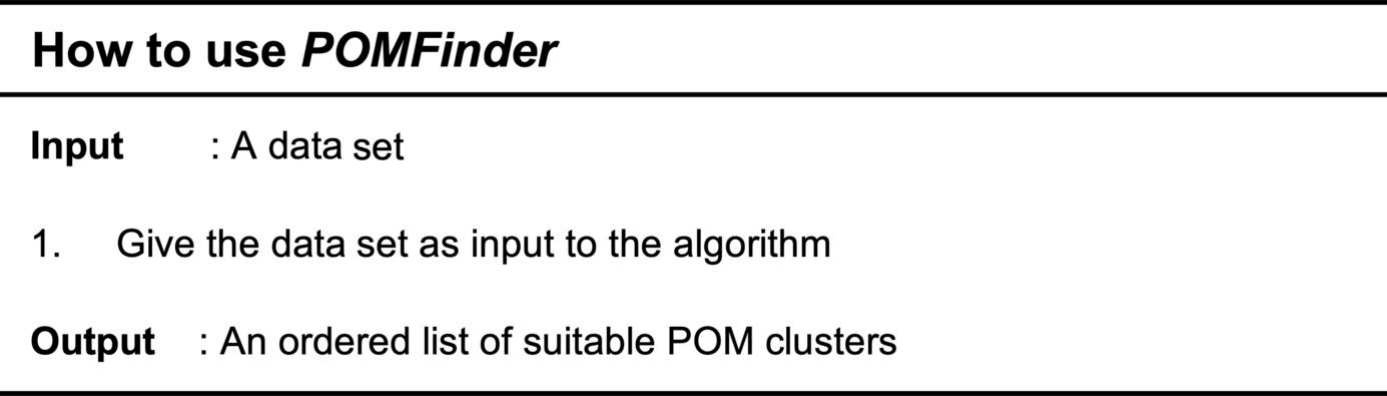
Pseudo-code describing how to use *POMFinder*. The data set is simply given as input to *POMFinder*, which outputs an ordered list of suitable POM clusters from which a few can be fitted to the data set.

*POMFinder* is a simple tool to use since everything is fully automated. As seen in Fig. 3 ▸, one simply provides a data set as input to *POMFinder*, and it will return a list of likely structures as output. The input here is a PDF but it can, in principle, be any data that can be modelled using an atomistic model and represented in a tabular data format. As *POMFinder* is designed for predicting single-phase POMs from their corresponding PDFs, it will be challenged if confronted with PDFs obtained from multi-phase cluster systems or from crystalline structures. The output will be given in the *XYZ* format providing the elements and coordinates of all the atoms in the structure.

## 4. Results and discussion

### 4.1. Identification of POM structures from experimental PDFs

We start by demonstrating the power of *POMFinder* on an experimental PDF from a 0.05 *M* aqueous solution of ammonium metatungstate hydrate, (NH_4_)_6_[H_2_W_12_O_40_]·*x*H_2_O, which is known to yield [H_2_W_12_O_40_]^6−^ ions with the α-Keggin structure (Juelsholt *et al.*, 2019 ▸). The data were collected on the DanMAX beamline (MAX IV, Lund, Sweden) using a wavelength of λ = 0.3542 Å, achieving a *Q*_max_ of 20 Å^−1^. The acquisition time for the total scattering data set was 15 min. Keggin structures [Fig. 2 ▸(*b*)] have the chemical composition [*XM*_12_O_40_]^*n*−^, where *X* is a tetrahedrally coordinated cationic central atom in the middle of the cluster or one to three H^+^ ions, *M* is the metal atom of the cluster, and *n* is the negative charge of the cluster. Keggin clusters are divided into five rotational isomers with increasing degrees of edge sharing, namely α, β, γ, δ and ɛ (Gumerova & Rompel, 2020 ▸; Jeannin, 1998 ▸; Sartzi *et al.*, 2015 ▸), although the δ isomer is not present in our POM database.

**Figure 4:**
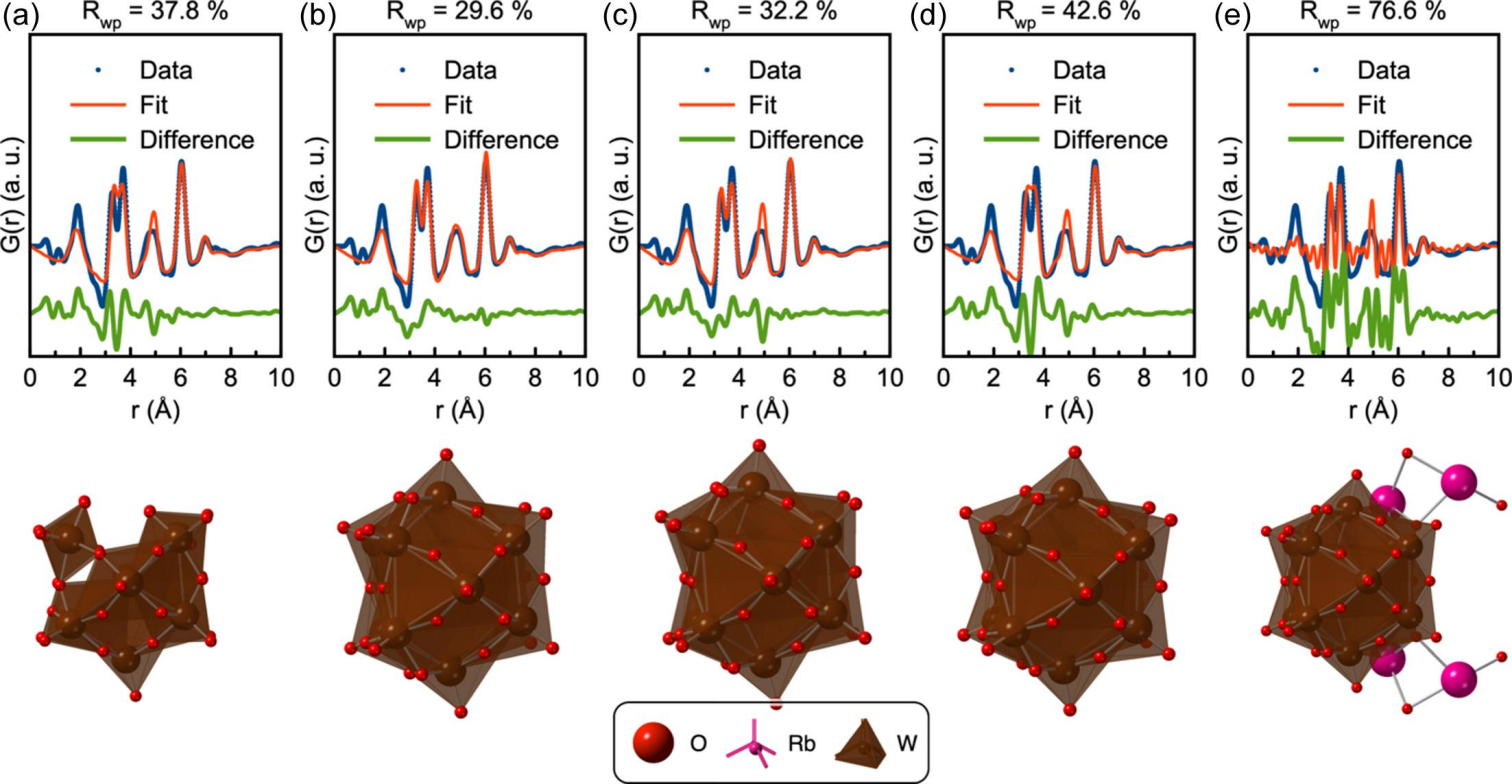
*POMFinder*’s top five predictions on experimental high-quality X-ray PDF data. Comparisons of the PDF obtained from the 0.05 *M* ammonium metatungstate solution and the fitted PDF of (*a*) a W_11_O_35_ Keggin-based fragment from the dimeric K_5.5_Na_7_Nd[SiW_11_O_39_ (H_2_O)]_2_(CH_3_COO)_2_(H_2_O)_10_ complex (Saini *et al.*, 2014 ▸), (*b*) a W_12_O_36_ fragment from the K_5_H(CoW_12_O_40_) (H_2_O)_15_ crystal (Glass *et al.*, 2014 ▸), (*c*) a W_12_O_40_ fragment from an ionic crystal structure of [Al_13_O_4_(OH)_24_(H_2_O)_12_](H_2_W_12_O_40_)(OH)(H_2_O)_23.12_ (Son *et al.*, 2003 ▸), (*d*) a W_12_O_36_ fragment from the porous inorganic structure of the formula K_2_NaH_2_(BW_12_O_40_)(H_2_O)_12_ (Han *et al.*, 2012 ▸) and (*e*) a W_12_Rb_4_BO_43_ fragment from another ionic crystal, Rb_4_[Cr_3_O(OOCH)_6_(H_2_O)_3_(BW_12_O_40_)](H_2_O)_16_ (Uchida *et al.*, 2006 ▸). W is shown in brown, O in red and Rb in pink. Refinement parameters are reported in Section D in the supporting information.

When the experimental PDF is given as input to *POMFinder*, the output is an ordered list of how probable it is that the PDF originates from each of the 443 POM structures in the POM database. The first five entries of the list are given in Section D in the supporting information, along with the probabilities assigned by *POMFinder*. The list clearly shows a dominance of structures with W_11–12_O_35–43_ composition which correspond to Keggin fragments. The five structures with the highest probability assigned by *POMFinder* are shown in Fig. 4 ▸, along with the fits to the experimental PDF. The first four candidate structures fit the PDF reasonably well. The best candidate, Fig. 4 ▸(*b*), with an *R*_wp_ value of 29.6%, is an α-Keggin structure. All the other structures are also α-Keggin structures or fragments.

### 4.2. Using *POMFinder* on fast acquisition data sets with a lower *Q*_max_

**Figure 5:**
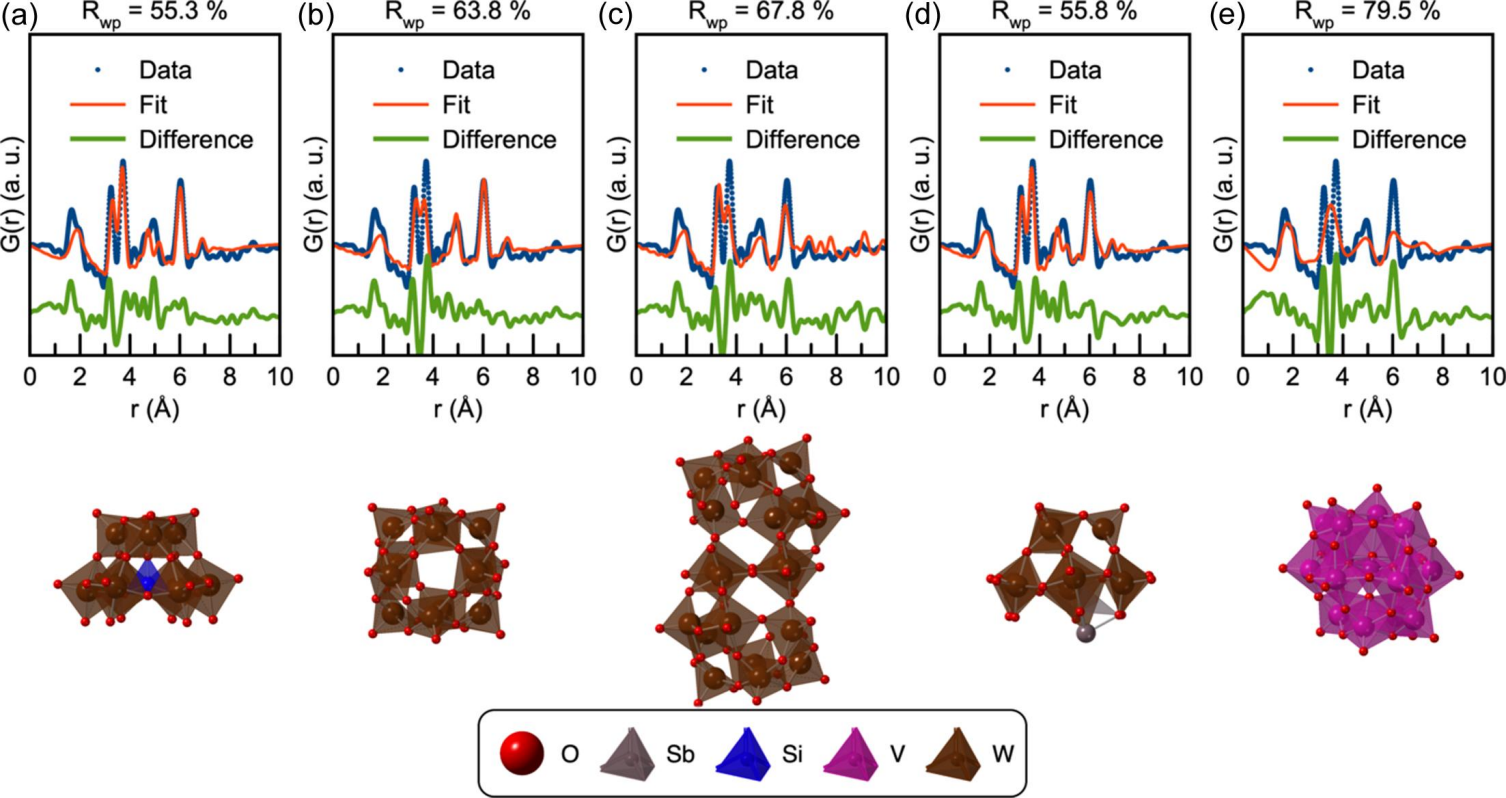
*POMFinder*’s top five predictions on experimental fast-acquisition X-ray PDF data. Comparisons of the PDF from a 0.05 *M* solution of ammonium metatungstate in oleylamine with (*a*) a W_9_SiO_34_ fragment from a Keggin-based Na_2_[C(NH_2_)_3_]_2_[[(CH_3_)_2_Sn(H_2_O)]_3_(*A*-α-SiW_9_O_34_)]·10H_2_O crystal (Piedra-Garza *et al.*, 2009 ▸), (*b*) a W_12_O_36_ fragment from the crystal structure of a porous framework based on Keggin polyoxoanions, K_2_NaH_2_[BW_12_O_40_]·12H_2_O (Han *et al.*, 2012 ▸), (*c*) a W_20_O_64_ fragment from a pseudo-Keggin-based crystal with chemical composition H_2−*x*_Bi_2_W_20_O_70_(HWO_3_) (Patrut *et al.*, 2010 ▸), (*d*) an SbW_9_O_30_ fragment from a K_11_[Sb_3_(SiW_9_O_34_)_2_]·31H_2_O crystal structure (Assran *et al.*, 2012 ▸) and (*e*) a V_15_O_42_ fragment from the bicapped Keggin structure (TMA)_3_H_6_V^V^_15_0_42_·2.5H_2_0 (TMA = tetra­methyl­ammonium) (Hou *et al.*, 1993 ▸). W is shown in brown, Sb in grey, O in red, Si in blue and V in pink. Refinement parameters are reported in Section D in the supporting information.

Having established that *POMFinder* can identify a POM structure from a high-quality experimental PDF, we are interested in examining the use of *POMFinder* for data acquired with a fast time resolution, as is the case for *in situ* data. X-ray total scattering with PDF analysis is a powerful technique to study the formation of *e.g.* oxides, and it has previously been shown that POM structures can play an important role in their formation (Skjaervø *et al.*, 2023 ▸; Juelsholt *et al.*, 2019 ▸; Bøjesen *et al.*, 2016 ▸; Saha *et al.*, 2014 ▸). Therefore, we tested *POMFinder* on fast-acquisition experimental PDFs with 2 s time resolution from the 0.05 *M* solution of ammonium metatungstate. The data quality for this data set only allows a *Q*_max_ of 16 Å^−1^. The data are the same as reported by Juelsholt *et al.* (2019 ▸) on the formation of tungsten oxide. The experimental PDF of the ammonium metatungstate solution (Fig. 5 ▸) shows a small structure with PDF peaks up to about 7 Å. When inputting the PDF to *POMFinder*, we again obtain an ordered list of possible structures, with the best five listed in Section D in the supporting information. Fig. 5 ▸ shows the fit of the five best predictions on the experimental PDF. The best fitting POM fragments, Figs. 5 ▸(*a*) and 5 ▸(*d*), are lacunary α-Keggin structures with three out of four triads. The second structure, Fig. 5 ▸(*b*), also reasonably fits the experimental PDF with an α-Keggin structure. In contrast, the structures in both Figs. 5 ▸(*c*) and 5 ▸(*e*) are too large to describe the experimental PDF well. Nevertheless, using *POMFinder* we can identify main motifs and thus determine a good model from fast-acquisition PDFs.

**Figure 6:**
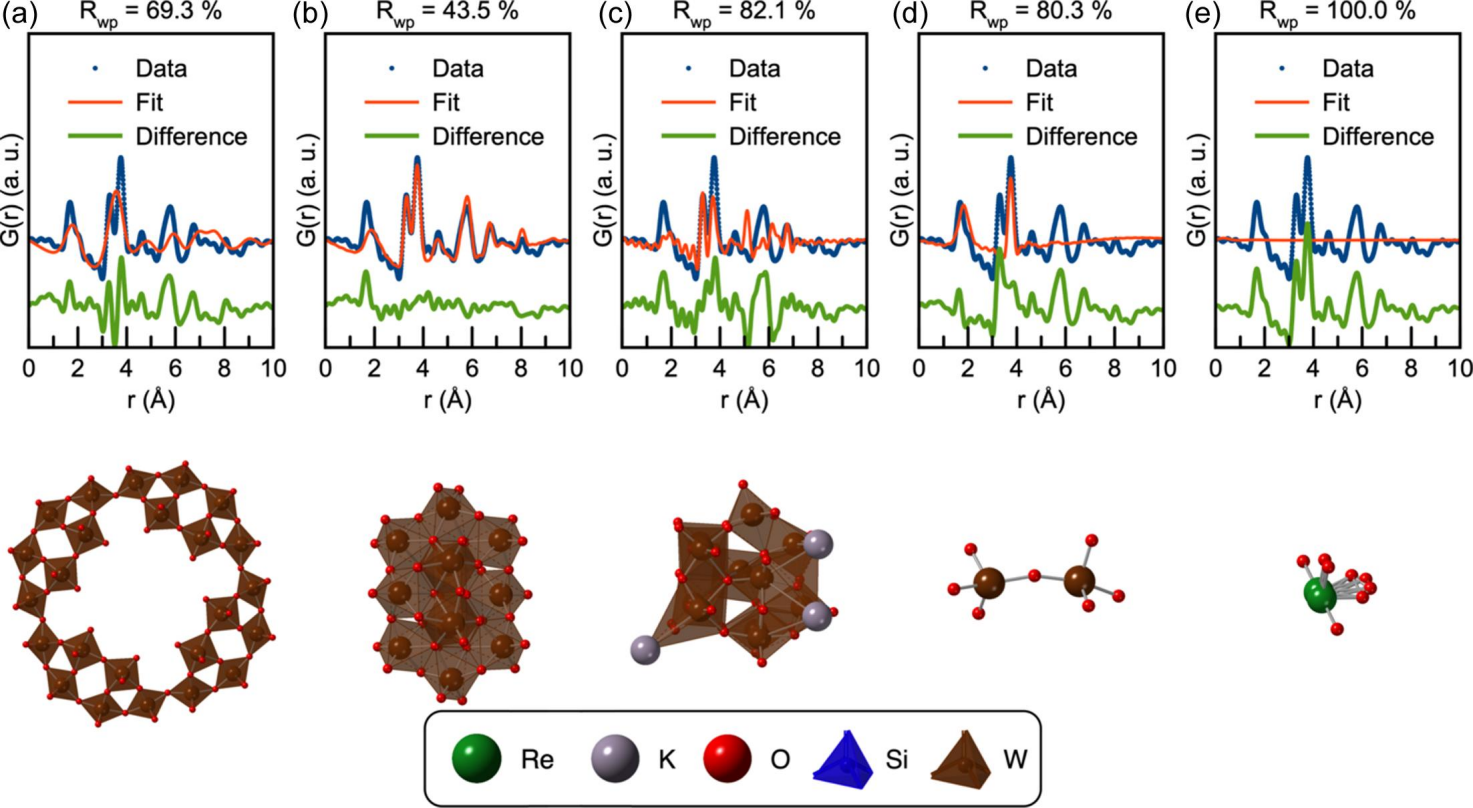
*POMFinder*’s top five predictions on experimental fast-acquisition X-ray PDF data. Comparisons of the PDF from a 0.05 *M* solution of ammonium metatungstate in oleylamine heated to 200°C for 4 min and the calculated PDF of (*a*) a W_48_O_152_ fragment from the polyanion K_26.5_Li_9.5_[H_4_As_8_W_48_O_184_]·90H_2_O (Mbomekallé *et al.*, 2014 ▸), (*b*) a W_12_O_42_ fragment from the acidic sodium polytungstate Na_5_[H_7_W_12_O_42_]·20H_2_O (Redrup & Weller, 2009 ▸), (*c*) a W_11_K_3_O_38_ fragment from the crystal structure K_6_H_4_W_11_O_38_·H_2_O (Lehmann & Fuchs, 1988 ▸), (*d*) a W_2_O_7_ fragment from the crystal structure of Bi_2_W_2_O_9_ (Champarnaud-Mesjard *et al.*, 1999 ▸) and (*e*) an Re_2_O_8_ fragment from the crystal structure Bi_28_Re_2_O_49_ (Crumpton *et al.*, 2005 ▸). W is shown in brown, K in grey, O in red, Si in blue and Re in green. Refinement parameters are reported in Section D in the supporting information.

As discussed by Juelsholt *et al.* (2019 ▸), another cluster appears when heating the 0.05 *M* solution of ammonium metatungstate in oleyl­amine to 200°C. The experimental PDF after *ca* 4 min of heating is shown in Fig. 6 ▸. When inputting the PDF to *POMFinder*, we again obtain an ordered list of possible structures, with the best five listed in Section D in the supporting information. Figs. 6 ▸(*a*)–6 ▸(*e*) show the first five structures suggested by *POMFinder* and their fits to the PDF. The second prediction, Fig. 6 ▸(*b*), is the only POM fragment that reasonably fits the experimental PDF. This is a paratungstate POM, which agrees with the conclusion reached by Juelsholt *et al.* (2019 ▸). *POMFinder* is thus very well suited for analysis of *in situ* data where small structural changes in the cluster structure are observed. We attempted to analyse the entire *in situ* data set from Juelsholt *et al.* (2019 ▸), comprising 1022 PDFs. This analysis was completed in 66.5 s using a standard laptop equipped with an Intel Core i7-8665U CPU at 1.9/2.11 GHz. *POMFinder* performs well for the stages in the *in situ* data set where only one cluster is present. However, many of the PDFs in the time-resolved data set contain signals from multiple cluster species. Here, *POMFinder* is challenged, as these types of data go beyond the training set used. At this point, *POMFinder* thus cannot be used for identifying suitable POM clusters for PDFs obtained from multiple coexisting POMs. This challenge could possibly be overcome by combining the use of *POMFinder* with *e.g.* principal component analysis or negative matrix factorization, which potentially could separate the signals from each POM in the PDF.

### 4.3. Rationalizing *POMFinder*’s predictions using SHAP values

**Figure 7:**
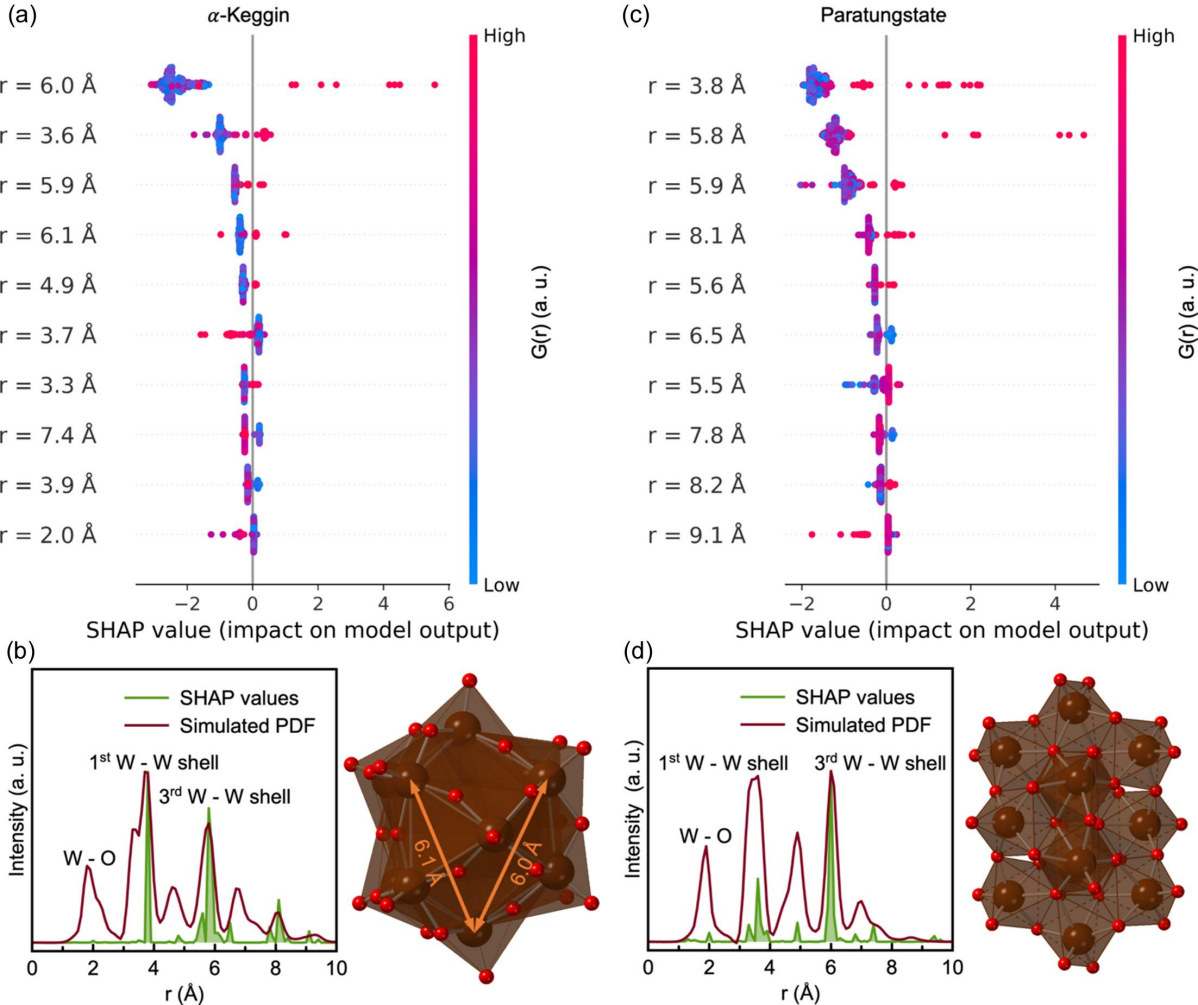
SHAP analysis of *POMFinder* on experimental PDFs. (*a*) and (*c*) For every PDF in the test set, SHAP values are calculated for all PDF intensities [*G*(*r*) values], indicated with red for peaks and blue for low intensities. The *r* values of the PDF intensity are shown as labels. In panel (*a*), only the impact of predicting the α-Keggin cluster is shown, while (*c*) shows the impact of predicting the paratungstate cluster. (*b*) and (*d*) Histograms of absolute SHAP values for each PDF intensity plotted versus the *r* values on top of the PDFs of (*b*) the α-Keggin cluster and (*d*) the paratungstate cluster. W is shown in brown and O in red.

The above results have established that *POMFinder* can identify the POM structure present in solutions from experimental PDFs, yet it is not clear on what *POMFinder* bases its predictions. To obtain this understanding, we use SHAP analysis. SHAP is a feature importance measure which yields information about how the ML model exploits the individual features in the input data to make its predictions. Here, the features are *Q*_min_, *Q*_max_, *Q*_damp_ and *G*(*r*) values for *r* values between 0.0 and 10.0 Å with a step size of 0.1 Å. A SHAP value is calculated for each feature for each PDF in the training set. The amplitude of the calculated SHAP value for a given feature provides information about how important the feature is, while the sign of the SHAP value tells whether the feature is confirming or disqualifying the specific structure as a match to the data set. Figs. 7 ▸(*a*) and 7 ▸(*c*) show a SHAP analysis of the two fast-acquisition PDFs discussed above, predicting the α-Keggin and the paratungstate cluster, respectively. The top of the figure shows the SHAP values of the most important features, *i.e.* those that give the highest amplitude of SHAP values. The value of the features, in this case the *G*(*r*) intensity, is indicated by colour: high PDF intensities [*G*(*r*) values] in the PDFs in the training set are represented in red, while low *G*(*r*) values are coloured blue. For the α-Keggin cluster, the SHAP analysis shows that the two most important features are the *G*(*r*) values at *r* = 6.0 Å and *r* = 3.6 Å. When inspecting the PDF and POM structures, these *r* values correspond to two W—W distances, as indicated in the structure drawing in Fig. 7 ▸(*b*). This means that *POMFinder* bases its predictions strongly on PDF peaks arising from W—W distances. W has a higher X-ray scattering power compared with O (W has 74 electrons, whereas O has eight), and W—W peaks are thus much more prominent in X-ray PDFs compared with W—O or O—O peaks (Prince, 2004 ▸). For paratungstate, we observe the same trend [Figs. 7 ▸(*c*) and 7 ▸(*d*)]. We conclude that *POMFinder* pre­dominantly bases its predictions on the intensities of the PDF peaks describing the first and third metal–metal shells for these two structures.

Instead of using SHAP to explain how *POMFinder* makes its predictions on individual PDFs, it is possible to get a global explanation by calculating an average of all the absolute SHAP values from the 443 POM structures in the POM structure database (shown in Section E in the supporting information). This analysis shows that the average absolute SHAP value for the *Q*_min_, *Q*_max_ and *Q*_damp_ values is insignificant, meaning that *POMFinder* is not sensitive to the provided user input of *Q*_min_, *Q*_max_ and *Q*_damp_ in the ranges used for training *POMFinder*. The average absolute SHAP value for *G*(*r*) values in the *r* = 0–1 Å range is 0 since they are fixed to *G*(*r* < 1 Å) = 0. However, the rest of the *G*(*r*) values all have some contribution to the prediction of *POMFinder*. In particular, the *G*(*r*) values corresponding to PDF peaks for *M*—O distances (∼2.0 Å) and the first and third metal–metal distances (∼3.3 and ∼6.2 Å, respectively) are important for *POMFinder*’s predictions.

**Figure 8:**
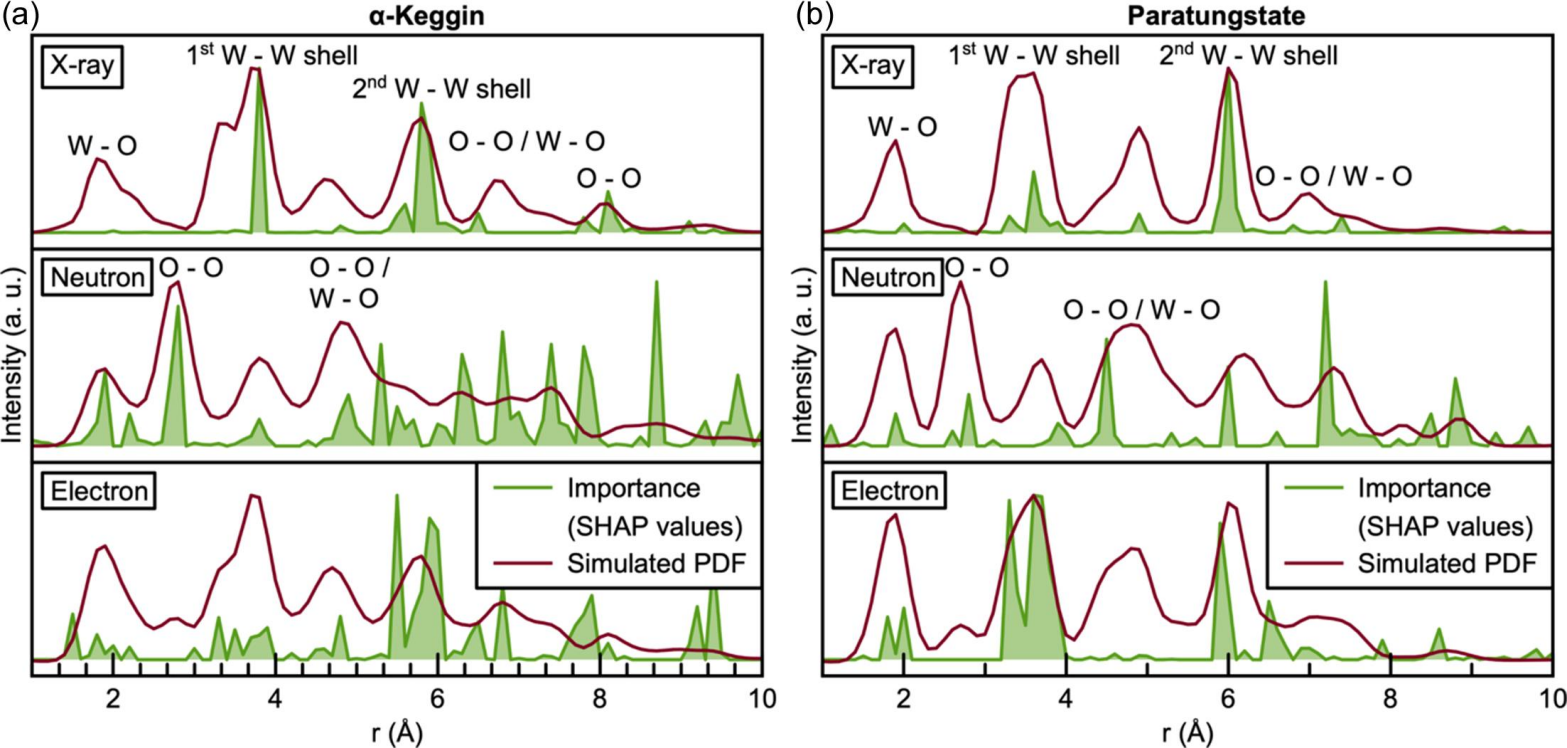
Analysis of the influence of the scattering probe on *POMFinder*’s predictions. (Top) The X-ray, (middle) the neutron and (bottom) the electron PDFs are plotted on top of a measure (SHAP values) of how important each data point in the PDF is for *POMFinder* to make its prediction on (*a*) the α-Keggin cluster and (*b*) the paratungstate cluster.

To confirm that the predictions from *POMFinder* relate to the scattering power of the elements, we conducted the same SHAP analysis on simulated nPDF and ePDF data. From the same POM database, we first simulated 100 nPDFs and ePDFs from each POM structure in our database with different *Q*_min_, *Q*_max_, *Q*_damp_ and atomic displacement parameters, trained a GBDT model using a 98:1:1 training:validation:test set split, and then applied SHAP analysis to investigate the results. Fig. 8 ▸ shows the SHAP values for each feature in *POMFinder* when trained on the simulated xPDF, nPDF and ePDF data compared with their relative simulated PDFs.

When *POMFinder* is trained on nPDFs, the SHAP value is high for features corresponding to O—O peaks and W—O peaks. The neutron scattering lengths of W and O are 4.9 and 5.8 fm, respectively (Prince, 2004 ▸). This means that *POMFinder* bases its predictions on the O—O and W—O peaks when trained on nPDFs, rather than on the W—W peaks as seen for xPDFs as discussed above. This is probably due to the comparable neutron scattering lengths of W and O in contrast to the scattering contrast between W and O in xPDF and ePDF experiments. As expected, *POMFinder* primarily bases its predictions on the W—W distances when trained on ePDFs (electron scattering factors: W 12.5 Å, O 2.0 Å; Prince, 2004 ▸), but it gives higher weights to the O atoms than for xPDF. We thus see a clear trend between the scattering power of the element and the reasoning of *POMFinder*. A similar global analysis of all structures in our POM database is shown in Section E in the supporting information, which provides comparable results. We therefore hypothesize that *POMFinder* learns about the scattering contrast of different elements when predicting which POM fragment a PDF matches.

**Figure 9:**
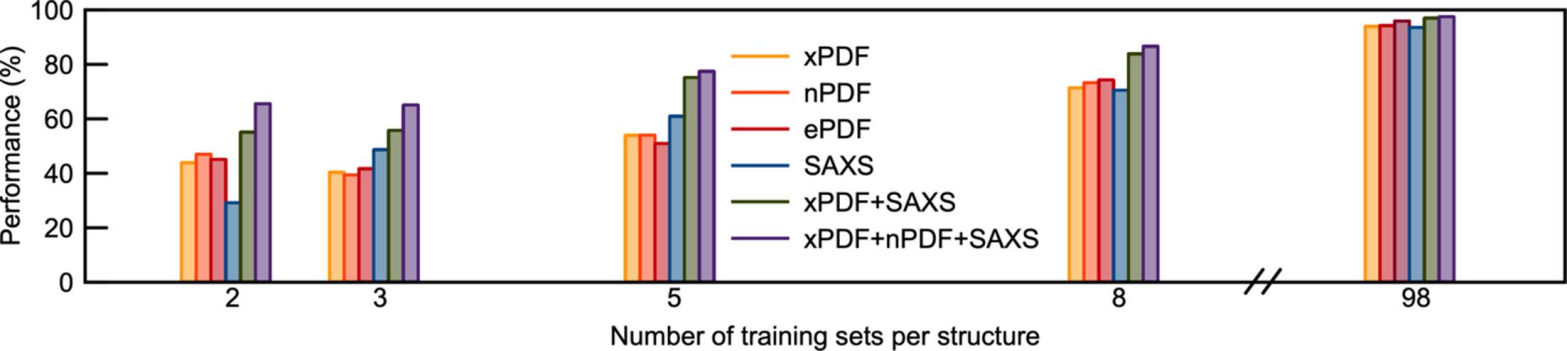
The performance of the model trained with various simulated data sets and different numbers of data sets per structure. Section F in the supporting information lists the mean and standard deviation based on five iterations where the model was trained on different simulated PDFs and predictions were made on the same test set.

Fig. 9 ▸ shows the performance of *POMFinder* on the test set when *POMFinder* is trained using splits of 2:1:1, 3:1:1, 5:1:1, 8:1:1 and 98:1:1 PDFs per POM structure. Unsurprisingly, the performance (defined as the accuracy of the model on the test set) of *POMFinder* increases when trained on more data. Generally, *POMFinder* performs comparably when trained on xPDF, nPDF and ePDF data, as seen in Fig. 9 ▸.

### 4.4. Combination with data from other techniques

It has previously been shown that combined modelling of data from multiple scattering techniques can provide more robust results than separately modelling data from the individual scattering techniques (Anker *et al.*, 2021 ▸; Juhás *et al.*, 2015 ▸; Farrow *et al.*, 2014 ▸; Farrow & Billinge, 2009 ▸; Tucker *et al.*, 2007 ▸; Krayzman *et al.*, 2008 ▸). However, it is a cumbersome process to do combined modelling of data from multiple scattering techniques using a least-squares approach, and it can be challenging to weight the contribution from each data set (Anker *et al.*, 2021 ▸; Juhás *et al.*, 2015 ▸; Krayzman *et al.*, 2008 ▸; Terban & Billinge, 2022 ▸). We hypothesize that this problem can be overcome with ML methods, and here we take the first steps to extend *POMFinder* to combined data sets. Specifically, we train *POMFinder* on a combination of xPDF/SAXS and a combination of xPDF/SAXS/nPDF data. We do not weight the data sets. The SAXS simulations provide information on the size and shape of the POM clusters and are thus highly complementary to the PDF data discussed above. Details of the SAXS simulations are given in Section B in the supporting information.

The results on performance are given in Fig. 9 ▸, where we observe that when combining information from PDF and SAXS experiments, the performance increases, especially when using small training sets where *POMFinder* is challenged when using data from only one technique. This example demonstrates that *POMFinder* can easily be extended to identify a structure from combined data sets and that combining information from various data sets provides a higher performance on a test set.

## 5. Conclusions

We have demonstrated how our tree-based ML classifier, *POMFinder*, can screen a POM structure database to identify structural candidates for the modelling of PDF data. Instead of using the traditional approach in scattering data analysis, where PDFs from all POM clusters in the database are fitted to the data through a least-squares refinement, we have shown that *POMFinder* can be used first to narrow down the field of candidate structures very quickly to five POM clusters, which can then be analysed further.

The POM database was made by cutting out clusters from the COD and ICSD databases following appropriate chemical restraints for POM structures. A GBDT model, *XGBoost*, was trained on simulated X-ray PDF data to classify the POM clusters with an accuracy of 94.0% on simulated PDFs. *POMFinder* also performs well on experimental data, including *in situ* data collected with a fast acquisition time. This ultrafast method allows *e.g.* visualizing the structural model in three dimensions while collecting data.

Using SHAP analysis, we have shown that *POMFinder* bases its predictions on trends comparable to the scattering contrast of the elements in the clusters.

Finally, we have shown that, in contrast to conventional complex modelling refinement methods, ML offers a promising and more flexible modelling framework for structure identification from combined data sets as it is not necessary to weight the data contributions (Anker *et al.*, 2021 ▸; Juhás *et al.*, 2015 ▸; Krayzman *et al.*, 2008 ▸).

*POMFinder* is open source, and the method can be directly applied by users without prior ML knowledge to characterize POM clusters.

*POMFinder* can be extended to include more types of chemical systems by extending the structural database used to generate the training data. In this project, we have focused on screening a database of POM fragments. However, the ultimate goal is to include any cluster fragment from the databases of known crystal structures, such as COD and ICSD which have more than 600 000 entries between them. The approach used for *POMFinder* can also be extended to analyse data from other scattering and spectroscopy techniques. We thus see *POMFinder* as a proof of concept, showing how a database of known structures can quickly be screened for analysis of *e.g.* scattering data using simple explainable ML methods.

## 6. Data availability

The database of POM clusters and the code used to train *POMFinder* is available at https://zenodo.org/records/10055030. *POMFinder* is available on GitHub at https://github.com/AndySAnker/POMFinder/. A web app to use *POMFinder* is available at https://huggingface.co/spaces/AndySAnker/POMFinder.

## Supplementary Material

Supporting information. DOI: 10.1107/S1600576723010014/in5097sup1.pdf
