# Supplementary material for: POMFinder: identifying polyoxometallate cluster structures from pair distribution function data using explainable machine learning
Source: J Appl Crystallogr. 2024 Feb 1;57(Pt 1):34–43. doi: 10.1107/S1600576723010014 (PMC10840315; doi:10.1107/S1600576723010014)
Supplement: Supplementary file 1 [file j-57-00034-sup1.pdf]

JOURNAL OF APPLIED CRYSTALLOGRAPHY

Volume 57 (2024)

Supporting information for article:

POMFinder: Identifying polyoxometallate cluster structures from pair distribution function data using explainable machine learning

Andy S. Anker, Emil T. S. Kjaer, Mikkel Juelsholt and Kirsten M. Ø. Jensen

Supporting information for:

POMFinder: Identifying polyoxometalate cluster structures from pair distribution function data using explainable machine learning

*Andy S. Anker[1], Emil T. S. Kjaer[1], Mikkel Juelsholt,[1,2] and Kirsten M. Ø. Jensen[1]**

1: Department of Chemistry & Nano-Science Center, University of Copenhagen, 2100 Copenhagen Ø, Denmark

2: Department of Materials, University of Oxford, Parks Road, Oxford, OX1 3PH, UK

***Correspondence to kirsten@chem.ku.dk (KMØJ)**

Table of Contents

A: Determining if two POM fragments are identical 2

B: Simulation parameters for the simulated data sets used to train POMFinder 3

C: Loss curve for the training of POMFinder 4

D: Refinement parameters for the fits shown in Figure 5-7 in the main paper 5

E: SHAP analysis 7

F: Performance versus the number of datasets 7

A: Determining if two polyoxometalate (POM) fragments are identical

Table S1: Simulation parameters for pair distribution functions (PDFs) used to determine if two clusters are similar. The isotropic atomic displacement parameters (ADP) have been set high to emphasise the general trends in the PDF and not the disorder.

R_{Range} = 0 – 30 Å
R_{step} = 0.1 Å
Q_{min} = 0.2 Å^{-1}
Q_{max} = 30 Å^{-1}
Q_{damp} = 0.01 Å^{-1}
ADP^{Biso} = 1 Å^{2}

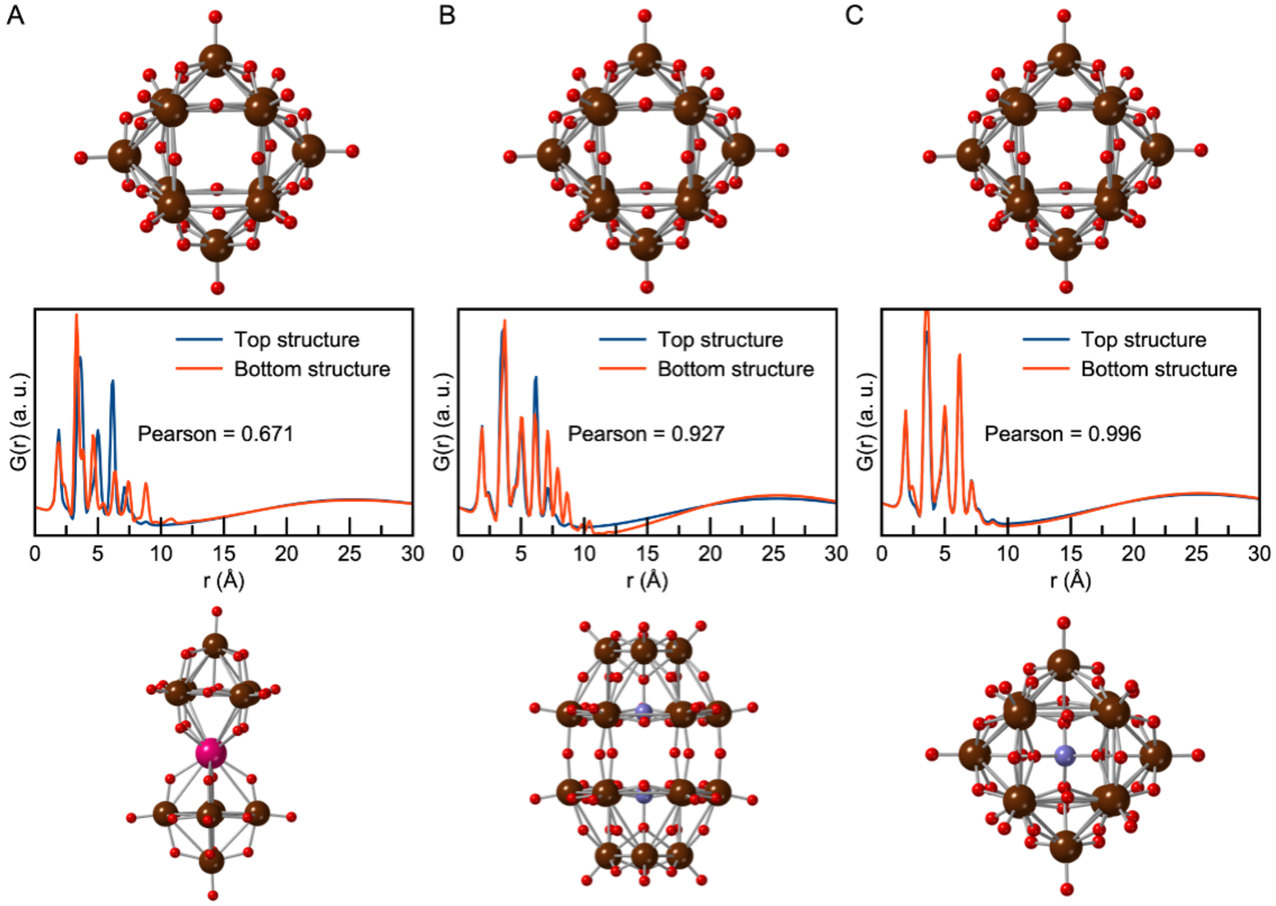

Figure S1: Three examples where the Pearson correlation coefficient is calculated between the simulated PDF of a $W_{12}O_{36}$ cluster and a simulated PDF of a A) $SmW_{10}O_{36}$ cluster, B) $As_2W_{18}O_{62}$ cluster and C) $PW_{12}O_{40}$ cluster.

B: Simulation parameters for the simulated data sets used to train POMFinder and normalisation

Table S2: Parameter range for the simulated data sets.[1] The simulated parameters were determined using hypercube sampling.[2]

PDF	SAXS
R_{Range} = 0 – 10 Å	Q_{Range} = 0 – 2 Å^{-1}
R_{step} = 0.1 Å	Background = 0 – 0.01

Q_{min} = 0 – 2 Å^{-1}	DebyeSumRmax = 10^7 Å
Q_{max} = 14 – 28 Å^{-1}	Gaussian Noise RMS = 0 – 0.01
Q_{damp} = 0.01 – 0.04 Å^{-1}	
ADP^{Biso} = 0 - 2 Å^2	

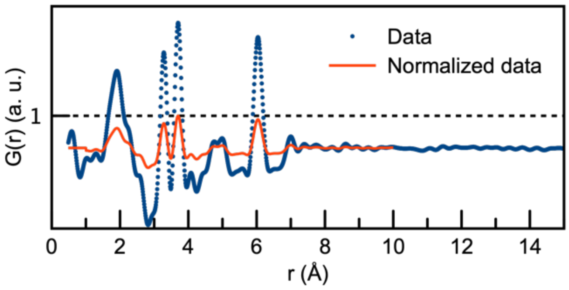

Figure S2: Normalisation of the PDF. Comparison of the experimental data set of a 0.05 M ammonium metatungstate hydrate, $(NH_4)_6[H_2W_{12}O_{40}]\cdot xH_2O$, solution before and after normalisation.

C: Loss curve for the training of POMFinder

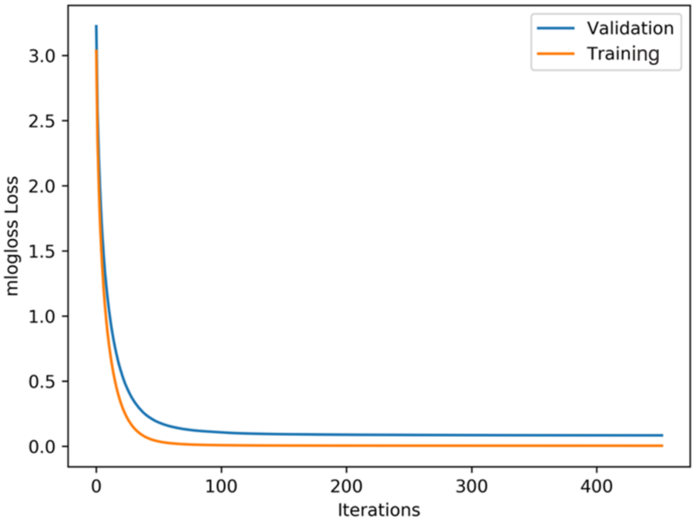

Figure S3: Loss curve for the training of the GBDT algorithm to predict which POM cluster a PDF matches. The training loss converges to zero, while the validation loss is slightly higher (overfitting). This means the model learns to predict perfectly on previously seen data sets, while it has minor disrepancies on unknown data. The mlogloss is defined elsewhere.[3]

D: Refinement parameters for the fits shown in Figure 4-6 in the main paper

Table S3: Refined parameters for the five best candidate POMs obtained from POMFinder for PDF data analysis. The structures are shown in Figure 4 in the main paper. The B_{iso} value for oxygen is set to 2 $Å^2$.

Rank	Probability [%]	Structure	Scale	Expansion/ contraction factor	B_{iso} (metal) [$Å^2$]
1st	82.8	A $W_{11}O_{35}$ Keggin-based fragment from the dimeric $K_{5.5}Na_7Nd(SiW_{11}O_{39}(H_2O))_2(CH_3COO)_2(H_2O)_{10}$ complex[4]	1.53	0.989	0.660
2nd	14.4	A $W_{12}O_{36}$ fragment from the $K_5H(CoW_{12}O_{40})(H_2O)_{15}$ crystal[5]	1.601	1.000	0.738
3rd	1.5	A $W_{12}O_{40}$ fragment from an ionic crystal structure[6] of $(Al_{13}O_4(OH)_{24}(H_2O)_{12})(H_2W_{12}O_{40})(OH)(H_2O)_{23.12}$	1.505	1.003	0.788
4th	1.0	A $W_{12}O_{36}$ fragment from the porous inorganic of the form $K_2NaH_2(BW_{12}O_{40})(H_2O)_{12}$[7]	1.414	1.004	0.822
5th	0.2	A $W_{12}Rb_4BO_{43}$ fragment from another ionic crystal[8] $Rb_4(Cr_3O(OOCH)_6(H_2O)_3(BW_{12}O_{40})(H_2O)_{16}$	0.514	1.000	0.000

Table S4: Refined parameters for the five best candidate POMs obtained from POMFinder for analysis of rapid acquisition PDF data. The structures are shown in Figure 5 in the main paper. The B_{iso} value for oxygen is set to 2 $Å^2$.

Rank	Probability [%]	Structure	Scale	Expansion/ contraction factor	B_{iso} (metal) [$Å^2$]
1st	65.0	A W_9SiO_{34} fragment from a Keggin-based	1.086	0.994	0.395

		$Na_2[C(NH_2)_3]_2[\{(CH_3)_2Sn(H_2O)\}_3(A\text{-}\boldsymbol{\alpha}\text{-}SiW_9O_{34})]\cdot 10H_2O$ crystal[9]			
2nd	27.4	A $W_{12}O_{36}$ fragment from the crystal structure of a porous framework based on Keggin polyoxoanions, $K_2NaH_2[BW_{12}O_{40}]\cdot 12H_2O$[10]	0.848	1.001	0.498
3rd	1.6	A $W_{20}O_{64}$ fragment from a pseudo-Keggin based crystal[11] with chemical composition H(2-x)$Bi_2W_{20}O_{70}(HWO_3)$	1.053	0.956	0.373
4th	1.6	A SbW_9O_{30} fragment from a $K_{11}[Sb_3(SiW_9O_{34})_2]\cdot 31H_2O$ crystal structure[12]	1.228	0.983	0.325
5th	0.9	A $V_{15}O_{42}$ fragment from the bicapped Keggin structure[13] $(TMA)_3H_6V^{V}{}_{15}0_{42}\cdot 2.5H_20$ (TMA = tetramethylammonium)	1.830	0.952	2.174

Table S5: Refined parameters for the five best candidate POMs obtained from POMFinder for analysis of rapid acquisition PDF data. The structures are shown in Figure 6 in the main paper. The B_{iso} value for oxygen is set to 2 Å^2.

**Rank**	**Probability [%]**	**Structure**	**Scale**	**Expansion/ contraction factor**	**B_{iso} (metal) [Å^2]**
1st	80.7	A $W_{48}O_{152}$ fragment from the polyanion $K_{26.5}Li_{9.5}[H_4As_8W_{48}O_{184}]\cdot 90H_2O$[14]	1.883	0.957	3.284
2nd	8.6	A $W_{12}O_{42}$ fragment from the acidic sodium polytungstates[15] $Na_5[H_7W_{12}O_{42}]\cdot 20H_2O$	1.176	0.989	0.503
3rd	1.4	A $W_{11}K_3O_{38}$ fragment from the crystal structure[16] $K_6H_4W_{11}O_{38}\cdot H_2O$	0.602	1.001	0.000
4th	1.0	A W_2O_7 fragment from the crystal structure[17] of $Bi_2W_2O_9$	1.885	0.998	0.338
5th	0.9	A Re_2O_8 fragment from the crystal structure $Bi_{28}Re_2O_{49}$[18]	0	-	-

E: SHAP analysis

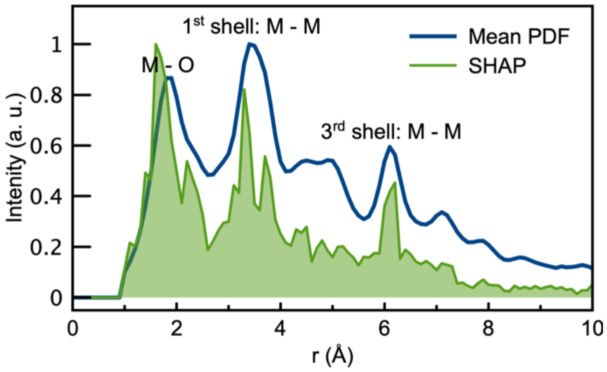

Figure S4: The average absolute SHAP value and PDF are calculated for all POM fragments in the database. Simulation parameters from section A in are used for the PDF simulation. When calculating the average PDF intensity, the intensity beyond the largest distance of the POM fragment is left out. The SHAP values for Q_{min}, Q_{max} and Q_{damp} are insignificant (Q_{min}: 0.003, Q_{max}: 0.003, Q_{damp}: 0.0006).

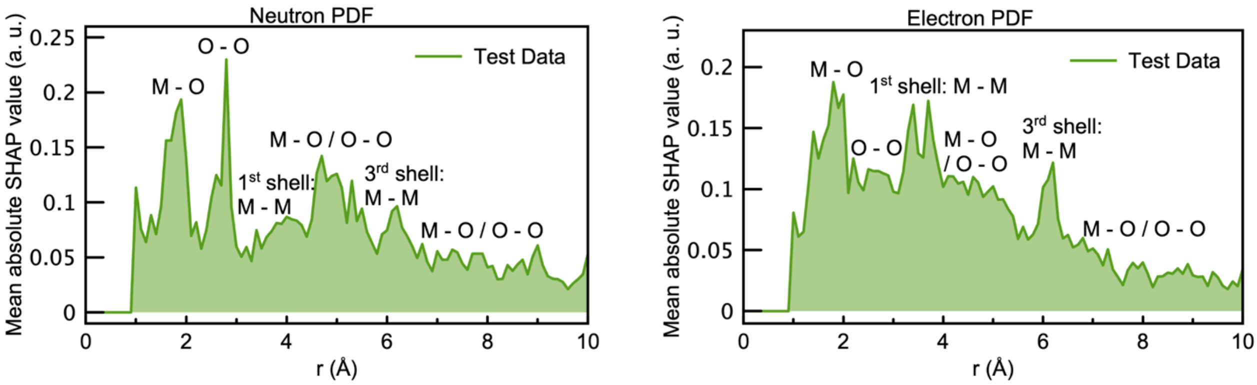

Figure S5: The average SHAP value over classes for the test set plotted for the r_{range} for A) nPDF data, B) ePDF data. Where The SHAP values for Q_{min}, Q_{max} and Q_{damp} are insignificant b) Q_{min}: 0.009, Q_{max}: 0.009, Q_{damp}: 0.006 and c) Q_{min}: 0.015, Q_{max}: 0.010, Q_{damp}: 0.007.

F: Performance versus the number of datasets

Table S6: The mean and standard deviation based on 5 iterations where the model has been trained on different simulated PDFs and predictions have been done on the same test set.

	**2**	**3**	**5**	**8**	**98**
**xPDF**	43.84 ± 0.09	40.32 ± 0.66	53.95 ± 0.00	71.38 ± 0.09	93.95 ± 0.16
**nPDF**	46.95 ±0.55	39.37 ± 0.27	54.00 ± 0.36	73.27 ± 0.31	94.27 ± 0.23

**ePDF**	45.10 ± 0.33	41.67 ± 0.51	50.93 ± 0.27	74.27 ± 0.00	95.98 ± 0.17
**xSAXS**	29.12 ± 3.32	48.67 ± 0.44	60.95 ± 0.00	70.56 ± 0.11	93.59 ± 0.11
**xPDF + xSAXS**	55.12 ± 0.22	55.76 ± 0.00	75.17 ± 0.00	83.88 ± 0.18	97.02 ± 0.17
**xPDF + nPDF + xSAXS**	65.55 ± 0.11	65.1 ± 0.11	77.47 ± 0.09	86.68 ± 0.00	97.52 ± 0.32

G: References

1. Juhás, P.; Farrow, C. L.; Yang, X.; Knox, K. R.; Billinge, S. J. L., Complex modeling: a strategy and software program for combining multiple information sources to solve ill posed structure and nanostructure inverse problems. *Acta Crystallogr. A* **2015,** *71* (Pt 6), 562-568.
2. Bouhlel, M. A.; Hwang, J. T.; Bartoli, N.; Lafage, R.; Morlier, J.; Martins, J. R. R. A., A Python surrogate modeling framework with derivatives. *Adv. Eng. Softw.* **2019,** *135*, 102662.
3. https://scikit-learn.org/stable/modules/generated/sklearn.metrics.log_loss.html.
4. Saini, M. K.; Gupta, R.; Parbhakar, S.; Kumar Mishra, A.; Mathur, R.; Hussain, F., Dimeric complexes of rare-earth substituted Keggin-type silicotungstates: syntheses, crystal structure and solid state properties. *RSC Adv.* **2014,** *4* (48), 25357-25364.
5. Glass, E. N.; Fielden, J.; Kaledin, A. L.; Musaev, D. G.; Lian, T.; Hill, C. L., Extending Metal-to-Polyoxometalate Charge Transfer Lifetimes: The Effect of Heterometal Location. *Chem. Eur. J.* **2014,** *20* (15), 4297-4307.
6. Son, J. H.; Kwon, Y.-U.; Han, O. H., New Ionic Crystals of Oppositely Charged Cluster Ions and Their Characterization. *Inorg. Chem.* **2003,** *42* (13), 4153-4159.
7. Han, X.-B.; Zhang, Z.-M.; Wang, Z.-S.; Zhang, H.; Duan, H.; Wang, E.-B., A 3D purely inorganic porous framework based on Keggin polyoxoanions. *Inorg. Chem. Commun.* **2012,** *18* (Complete), 47-49.
8. Uchida, S.; Kawamoto, R.; Mizuno, N., Recognition of Small Polar Molecules with an Ionic Crystal of α-Keggin-Type Polyoxometalate with a Macrocation. *Inorg. Chem.* **2006,** *45* (13), 5136-5144.
9. Piedra-Garza, L. F.; Reinoso, S.; Dickman, M. H.; Sanguineti, M. M.; Kortz, U., The first 3-dimensional assemblies of organotin-functionalized polyanions. *Dalton Trans.* **2009,** (31), 6231-6234.

10. Xin-Bao, H.; Zhi-Ming, Z.; Zhi-Shu, W.; Huan, Z.; Hui, D.; En-Bo, W., A 3D purely inorganic porous framework based on Keggin polyoxoanions. *Inorg. Chem. Commun.* **2012,** *18*, 47-49.

11. Patrut, A.; Bögge, H.; Forizs, E.; Rusu, D.; Lowy, D. A.; Margineanu, D.; Naumescu, A., Spectroscopic and crystal structure investigation of a new bismuth (III) containing polyoxometalate cluster. *Rev. Roum. Chim* **2010,** *55* (11-12), 865-870.

12. Assran, A. S.; Izarova, N. V.; Banerjee, A.; Rabie, U. M.; Abou-El-Wafa, M. H. M.; Kortz, U., The antimony(iii)-bridged heteropolyanion sandwich dimers $[Sb^{III}_3(A\text{-}\alpha\text{-}XW_9O_{34})_2]^{11-}$ (X = Si^{IV}, Ge^{IV}) and C-shaped double-sandwich $[Sb^{III}_6O_2(PW_6O_{26})(A\text{-}\alpha\text{-}PW_9O_{34})_2]^{15-}$. *Dalton Trans.* **2012,** *41* (33), 9914-9921.

13. Hou, D.; Hagen, K. S.; Hill, C. L., Pentadecavanadate, $V_{15}O_{42}^{9-}$, a new highly condensed fully oxidized isopolyvanadate with kinetic stability in water. *J. Chem. Soc., Chem. Commun.* **1993,** (4), 426-428.

14. Mbomekallé, I.-M.; Bassil, B. S.; Suchopar, A.; Keita, B.; Nadjo, L.; Ammam, M.; Haouas, M.; Taulelle, F.; Kortz, U., Improved Synthesis, Structure, and Solution Characterization of the Cyclic 48-Tungsto-8-Arsenate(V), $[H_4As_8W_{48}O_{184}]^{36-}$. *J. Cluster Sci.* **2014,** *25* (1), 277-285.

15. Redrup, K. V.; Weller, M. T., Hydrothermal routes to new sodium hydrogen polytungstates. *Dalton Trans.* **2009,** (23), 4468-4472.

16. Lehmann, T.; Fuchs, J., Struktur und Schwingungsspektrum des Kaliumundecawolframats $Kg_6H_4W_{11}O_{38}\cdot 11H_2O$ / Structure and Vibrational Spectrum of the Potassium Undecatungstate $K_6H_4WnO_{38}$ -11 H_2O. *Z. Naturforsch.* **1988,** *43* (1), 89-93.

17. Champarnaud-Mesjard, J.-C.; Frit, B.; Watanabe, A., Crystal structure of $Bi_2W_2O_9$, the n=2 member of the homologous series $(Bi_2O_2)B^{VI}{}_nO_{3n+1}$ of cation-deficient Aurivillius phases. *J. Mater. Chem.* **1999,** *9* (6), 1319-1322.

18. Crumpton, T. E.; Mosselmans, J. F. W.; Greaves, C., Structure and oxide ion conductivity in $Bi_{28}Re_2O_{49}$, a new bismuth rhenium oxide containing tetrahedral and octahedral Re(vii). *J. Mater. Chem.* **2005,** *15* (1), 164-167.
